# The effects of different inhalation therapies on less symptomatic chronic obstructive pulmonary disease patients in a Chinese population: a real-world study

**DOI:** 10.1080/07853890.2023.2192519

**Published:** 2023-03-29

**Authors:** Qing Song, Ling Lin, Aiyuan Zhou, Wei Cheng, Cong Liu, Yating Peng, Zijing Zhou, Yuqin Zeng, Dingding Deng, Dan Liu, Zhiping Yu, Yan Chen, Shan Cai, Ping Chen

**Affiliations:** a Department of Respiratory and Critical Care Medicine, The Second Xiangya Hospital, Central South University, Changsha, Hunan, China; b Research Unit of Respiratory Disease, Central South University, Changsha, Hunan, China; c Diagnosis and Treatment Center of Respiratory Disease, Central South University, Changsha, Hunan, China; d Center of Respiratory Medicine, Xiangya Hospital, Central South University, Changsha, Hunan, China; e Department of Respiratory Medicine, The First Attached Hospital of Shaoyang University, Shaoyang, Hunan, China; f Department of Respiratory, The Eighth Hospital in Changsha, Hunan, China; g Department of Respiratory, Longshan Hospital of Traditional Chinese Medicine, Hunan, China

**Keywords:** COPD, GOLD, less symptomatic group, inhalation therapy, exacerbation, mortality, pulmonary function

## Abstract

**Background:**

The Global Initiative for Chronic Obstructive Lung Disease (GOLD) document suggests that patients with chronic obstructive pulmonary disease (COPD) should be divided into a less symptomatic group. Moreover, single-inhaled drugs are recommended as initial inhalation therapy for them. However, many less symptomatic patients are provided double or triple-inhaled drugs as initial therapy in the real world. This study aimed to describe the inhalation prescriptions and compare the effects of different inhalation therapies on less symptomatic COPD patients.

**Patients and methods:**

This was an observational study. Stable COPD patients were recruited and divided into a less symptomatic group including Groups A and C based on the GOLD 2019 document. We collected the data of inhalation therapies prescriptions. Then, the patients were classified into long-acting muscarinic antagonist (LAMA), long-acting β2-agonist (LABA) + inhaled corticosteroid (ICS), LABA + LAMA, and LABA + LAMA + ICS groups. All the patients were followed up for 1 year to collect exacerbation and mortality data.

**Results:**

We found that only 45.4% of patients in Group A and 43.6% of patients in Group C received reasonable inhalation therapy in reference to the GOLD document. In addition, the LAMA group had a higher forced expiratory volume in one second (FEV1), FEV1%pred, FEV1/forced vital capacity and peak expiratory flow compared with LABA + ICS, LABA + LAMA and LABA + LAMA + ICS groups. However, we did not find any significant differences of exacerbation, hospitalization and mortality during the follow-up among different inhalation therapies groups on less symptomatic COPD patients.

**Conclusion:**

Over half of the less symptomatic patients received inhalation therapy that were inconsistent with the GOLD document recommendations in a Chinese population in the real world. In fact, the single inhaled drug of LAMA should be recommended and pulmonary function is not a good indicator for the choice of initial inhalation therapy in less symptomatic COPD patients.

**KEY MESSAGES**
- Over half of the less symptomatic COPD patients received inhalation therapy that were inconsistent with the GOLD document recommendations in a Chinese population in the real world.
- The clinicians should offer a single inhaled drug of LAMA to less symptomatic COPD patients and pulmonary function is not a good indicator for the choice of initial inhalation therapy.

## Introduction

Chronic obstructive pulmonary disease (COPD) is the most common chronic respiratory disease. It has high morbidity and mortality and exerts a huge burden on societies and healthy systems. Therefore, effective treatment is essential to mitigate the progression of the disease [1].

The Global Initiative for Chronic Obstructive Lung Disease (GOLD) recommends that patients with COPD be treated based on the best available scientific evidence; its recommendations are revised annually. Until the GOLD 2011 document, the patients with COPD were evaluated based on the COPD assessment test (CAT), modified medical research council (mMRC), exacerbation risk, and pulmonary function. Then, patients are classified into a less symptomatic group (including Groups A and C) and a more symptomatic group [2]. However, the GOLD 2017 document revised the assessment tool and separated pulmonary function to better guide therapy [3]. As currently recommended by the GOLD document, initial inhalation therapy should be a bronchodilator (short or long-acting) for Group A and a single long-acting muscarinic antagonist (LAMA) for Group C [4]. Subsequent escalation and/or de-escalation of pharmacological management should be based on an individualized assessment of symptoms and exacerbation risk. However, the current status of initial inhalation therapy for less symptomatic COPD patients in the real world and whether the clinician have adhered GOLD document recommendations was not completely clear.

In addition, the LAMA is the most commonly used bronchodilator. Previous studies have shown that LAMA is superior to long-acting β2-agonist (LABA) in reducing the number of exacerbations and hospitalizations. Furthermore, long-term use of a LAMA could improve the patient’s respiratory symptoms and health status [5–7]. Therefore, single LAMA has become the preferred initial inhalation therapy for less symptomatic patients with COPD. However, studies have shown that less than half of less symptomatic patients with COPD started with LAMA monotherapy or adhered GOLD document recommendation [8–10]. These findings indicate that most of the patients are not provided an initial inhalation therapy based on the GOLD document. Rather, they are offered LABA + inhaled corticosteroid (ICS), or LABA + LAMA, or LABA + LAMA + ICS, or other inhalation therapies. However, in the real-word, the basis on which clinicians choose an initial inhalation therapy for less symptomatic patients with COPD remains unclear.

Therefore, the purpose of this study was to describe the inhalation prescriptions and analyze the effects on clinical outcomes of different inhalation therapies among less symptomatic patients COPD in the real world and to determine whether the pulmonary function is a good indicator for the choice of initial inhalation therapy.

## Patients and methods

### Study participants

This was a real-world, observational study. All Chinese subjects were from the outpatient COPD database set up by the Second Xiangya Hospital of Center South University (ChiCTR-POC-17010431) (Website: http://120.77.177.175:9007/a/login) that includes the Second Xiangya Hospital of Central South University, the First Attached Hospital of Shaoyang University, the Eighth Hospital in Changsha and Longshan Hospital of Traditional Chinese Medicine (Hunan, China). The patients who diagnosed with COPD at their first hospital visit were enrolled in this study between January 2019 and February 2021 according to the GOLD 2019 document: the ratio of forced expiratory volume in 1s to forced vital capacity (FEV1/FVC) was < 0.70 after inhaling a bronchodilator. Patients with bronchiectasis; asthma; lung cancer; pneumonia; pulmonary fibrosis were excluded.

This study was conducted in accordance with the Declaration of Helsinki and approved by the Ethics Committee of the Second Xiangya Hospital of Central South University, the First Attached Hospital of Shaoyang University, the Eighth Hospital in Changsha and Longshan Hospital of Traditional Chinese Medicine (Hunan, China). All patients provided their informed consent.

### Determination of the sample size

We used PASS software (version 15.0.5) to calculate the sample size, with a proportion of less symptomatic patients with COPD of 0.3 based on a previous study (Song et al. 2021), a confidence level of 0.95, and a confidence interval width (two-sided) of 0.08. Considering a dropout rate 20%, the minimum sample size was 659.

### Study designed

According to the GOLD 2019 document, patients with COPD were assigned to the less symptomatic group including Groups A and C. Briefly, Group A shows 0 to 1 exacerbation per year, no hospitalization, a CAT scores <10, and/or an mMRC score of 0 to 1. Group C shows exacerbations ≥2 or hospitalization ≥1 per year, a CAT scores <10, and/or an mMRC score of 0 to 1 [4]. Then, the COPD patients were classified into the LAMA, LABA + ICS, LABA + LAMA, and LABA + LAMA + ICS groups based on the inhalation therapy prescriptions they received at their first hospital visit.

### Data collection and variable definition

Data including demographics, smoke history, pulmonary function, the GOLD grades, CAT, mMRC, Clinical COPD Questionnaire (CCQ), exacerbations and hospitalizations in the past year, and inhalation therapy prescriptions were collected. After the patients were followed-up for 1 year; the number of exacerbations, hospitalizations, and deaths was collected.

An exacerbation is a COPD progression that requires antibiotics, or oral corticosteroids, or hospitalization [4]. A current-smoker has had smoking exposure of ≥10 pack-years, while an ex-smoker has had ≥10 pack-years but has not smoked for more than 6 months [11]. The GOLD 2019 document defines grades 1–4 as follows: grade 1, FEV1 ≥ 80%pred; grade 2, FEV1 50–79%pred; grade 3, FEV1 30–49%pred; and grade 4, FEV1 < 30%pred [4]. LABA/ICS and LABA/LAMA mean the two drugs are in an inhaler.

### Consistency with GOLD recommendation

According to the GOLD 2019 document, the initial inhaled therapy for less symptomatic COPD patients including Group A and Group C is recommended as follows: Group A, A short- or a long-acting bronchodilator (including short-acting β2-agonist, short-acting muscarinic agonist, LAMA and LABA) and Group C, LAMA [4]. If not defined as inappropriate therapy or as a discrepancy with the GOLD document.

### Statistical analysis

SPSS 26.0 (IBM, Armonk, NY, USA) and Free Statistics software version 1.7.1 (Beijing, China) were used for the statistical analysis of the data. Continuous variables are expressed as mean ± standard deviation (SD) or median and interquartile range (IQR) as appropriate. Continuous variables with a normal distribution and homogeneity of variance were analyzed with analysis of variance; otherwise, non-parametric tests were used. The chi-square test or Fisher’s exact test was used to analyze categorical variables. The odds ratio (OR) and their 95% confidence intervals (CI) were calculated using multiple logistic regression. *p* < .05 was considered to be statistically significant.

## Results

### Demographics and inhalation therapy of total less symptomatic COPD patients

**Figure 1.**
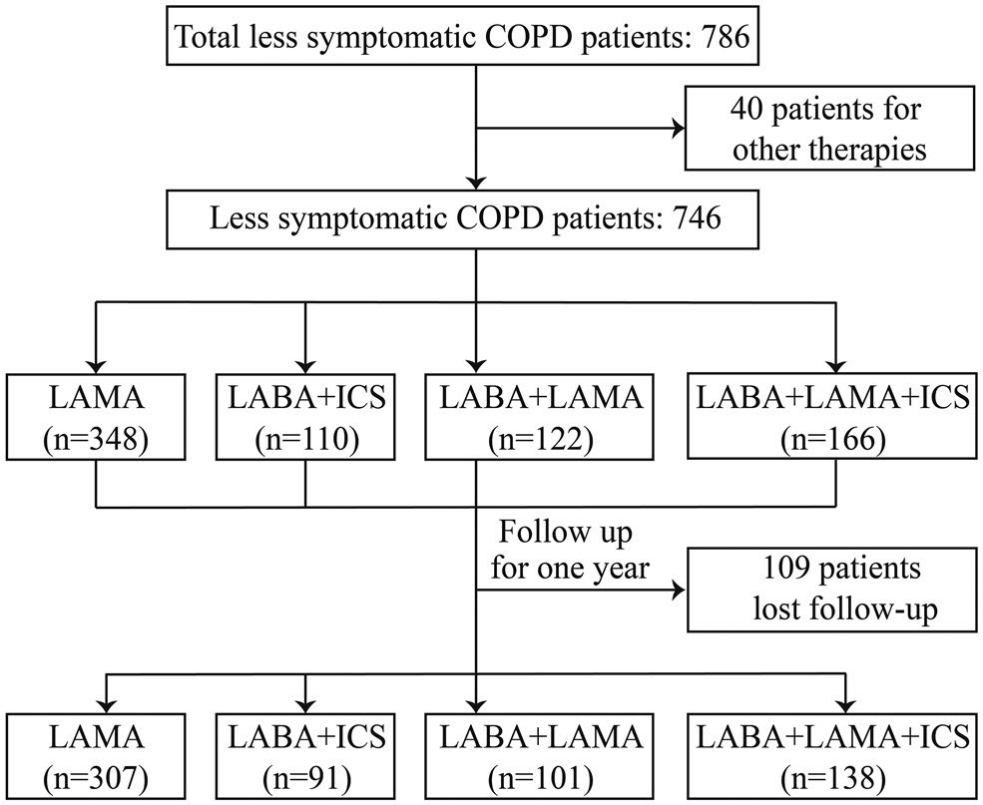
Flow chart. COPD: Chronic Obstructive Pulmonary Disease; ICS: Inhaled Corticosteroid; LAMA: Long-Acting Muscarinic Antagonist; LABA: Long-Acting β2-Agonist.

A total of 786 less symptomatic patients with COPD were enrolled in this study (Figure 1). The mean age was 63.2 ± 9.0 years and the majority were male (88.3%). The patients were assigned to Groups A (67.1%) and C (32.9%) (Table 1).

**Table 1.** Baseline characteristics of the total less symptomatic COPD patients.

Variables	Total (*n* = 786)
Age (years), (Mean ± SD)	63.2 ± 9.0
Sex, *n* (%)	
Male	694 (88.3)
Female	92 (11.7)
Education level, *n* (%)	
Primary school	287 (36.5)
Junior high school	278 (35.4)
High school	153 (19.5)
University	68 (8.6)
BMI (kg/m^2^), (Mean ± SD)	23.1 ± 3.3
Smoke history, *n* (%)	
Never-smoker	133 (17.9)
Ex-smoker	190 (24.2)
Current-smoker	463 (58.9)
Smoking, (pack/year) (Median, IQR)	39 (30)
Pulmonary function, (Mean ± SD)	
FEV1	1.6 ± 0.6
FEV1 %pred	64.8 ± 20.5
FVC	3.0 ± 0.8
FEV1/FVC	53.5 ± 11.1
PEF	4.4 ± 1.8
GOLD grades, *n* (%)	
1	172 (21.9)
2	416 (52.9)
3	176 (22.4)
4	22 (2.8)
Groups, *n* (%)	
A	527 (67.1)
C	259 (32.9)
CAT, (Mean ± SD)	9.1 ± 4.5
mMRC, (Median, IQR)	1 (1)
CCQ, (Mean ± SD)	16.3 ± 6.1
Exacerbations in the past year, (Median, IQR)	0 (1)
Exacerbations in the past year, *n* (%)	
0	449 (57.1)
1	203 (25.8)
≥2	134 (17.1)
Hospitalizations in the past year, (Median, IQR)	0 (0)
Hospitalizations in the past year, *n* (%)	
0	591 (75.2)
≥1	195 (24.8)

Abbreviations: BMI: Body Mass Index; COPD: Chronic Obstructive Pulmonary Disease; CAT: COPD Assessment Test; CCQ: Clinical COPD Questionnaire; FEV1: Forced Expiratory Volume in one second; FVC: Forced Vital Capacity; GOLD: Global Initiative for Chronic Obstructive Lung Disease; IQR: Interquartile Range; mMRC: modified Medical Research Council; PEF: Peak Expiratory Flow; SD: Standard Deviation.

Of the patients, the top inhalants used were LAMA (44.3%), LABA/ICS + LAMA (21.1%), LABA/LAMA (15.5%) and LABA/ICS (14.0%). Similar results were observed in Groups A and C. It was worth noting that only 45.4% of Group A patients and 43.6% of Group C patients were provided initial inhalation therapy adhered GOLD document recommendation in the real world (Table 2).

**Table 2.** Description of inhalation therapy.

Variables	Total (*n* = 786)	Group A (*n* = 527)	Group C (*n* = 259)	*p*- value
Single therapy, *n* (%)				
SABA	3 (0.4)	3 (0.57)	0 (0)	.555
SAMA	1 (0.13)	1 (0.19)	0 (0)	1.000
LAMA	348 (44.3)	235 (44.6)	113 (43.6)	.798
ICS	1 (0.13)	1 (0.19)	0 (0)	1.000
Double therapy, *n* (%)				
LABA/ICS	110 (14.0)	75 (14.23)	35 (13.5)	.785
LABA/LAMA	122 (15.5)	82 (15.6)	40 (15.4)	.966
Triple therapy, *n* (%)				
LABA/ICS + LAMA	166 (21.1)	105 (19.9)	61 (23.5)	.241
Other therapies, *n* (%)				
LAMA + SAMA	4 (0.5)	4 (0.76)	0 (0)	.309
LAMA + SABA	7 (0.9)	4 (0.76)	3 (1.2)	.690
SABA + SAMA	1 (0.13)	1 (0.19)	0 (0)	1.000
LAMA + SABA + SAMA	1 (0.13)	1 (0.19)	0 (0)	1.000
LAMA/LABA + SABA	1 (0.13)	1 (0.19)	0 (0)	1.000
LAMA/LABA + SAMA	5 (0.6)	3 (0.57)	2 (0.8)	.667
LABA/ICS + SABA	8 (1.0)	7 (1.3)	1 (0.4)	.283
LABA/ICS + SAMA	2 (0.25)	1 (0.19)	1 (0.4)	.551
LABA/ICS + LAMA + SABA	6 (0.8)	3 (0.57)	3 (1.2)	.402

LABA/ICS means LABA and ICS are in an inhaler. LAMA/LABA also means they are in an inhaler.

Abbreviations: ICS: Inhaled Corticosteroid; LAMA: Long-Acting Muscarinic Antagonist; LABA: Long-Acting β2-Agonist; SABA: Short-Acting β2-Agonist; SAMA: Short-Acting Muscarinic Agonist.

### The clinical characteristics of different inhalation therapies groups

To investigate the possible reasons on which clinicians choose an initial inhalation therapy for less symptomatic patients with COPD. We analyzed the clinical characteristics of less symptomatic patient treatment with LAMA (*n* = 348, 46.6%), LABA/ICS (*n* = 110, 14.7%), LABA/LAMA (*n* = 122, 16.4%), and LABA/ICS + LAMA (*n* = 166, 22.3%) which was the top used inhalants in the real world. As shown in Table 3, FEV1, FEV1%pred, FVC, FEV1/FVC, and peak expiratory flow (PEF) were higher in the LAMA group compared with the other groups (*p* < .05). In addition, the proportion of patients in GOLD grade 1 was higher in the LAMA group, while the proportion of patients in GOLD grades 3-4 was lower (*p* < .05). In addition, the CAT and CCQ scores in the LAMA group were lower than in the LABA + ICS and LABA + LAMA + ICS groups (*p* < .05).

**Table 3.** The clinical characteristics of different inhalation therapies on less symptomatic COPD patients.

Variables	LAMA (*n* = 348)	LABA + ICS (*n* = 110)	LABA + LAMA (*n* = 122)	LABA + LAMA + ICS (*n* = 166)	*p*-value
Age (years), (Mean ± SD)	62.7 ± 9.6	62.9 ± 9.4	63.4 ± 8.8	63.9 ± 8.0	.593
Sex, *n* (%)					.748
Male	303 (87.1)	96 (87.3)	107 (87.7)	150 (90.4)	
Female	45 (12.9)	14 (12.7)	15 (12.3)	16 (9.6)	
Education level, *n* (%)					.191
Primary school	140 (40.2)	33 (30.0)	44 (36.1)	56 (33.7)	
Junior high school	113 (32.5)	38 (34.5)	46 (37.7)	68 (41.0)	
High school	62 (17.8)	31 (28.2)	20 (16.4)	31 (18.7)	
University	33 (9.5)	8 (7.3)	12 (9.8)	11 (6.6)	
BMI (kg/m^2^), (Mean ± SD)	23.5 ± 3.1 #	22.9 ± 3.2	22.5 ± 3.4	23.0 ± 3.0	**.015**
Smoke history, *n* (%)					.052
Never-smoker	65 (18.7)	22 (20.0)	21 (17.2)	22 (13.3)	
Ex-smoker	68 (19.5)	28 (25.5)	34 (27.9)	54 (32.5)	
Current-smoker	215 (61.8)	60 (54.5)	67 (54.9)	90 (54.2)	
Smoke, (pack/year) (Median, IQR)	38.5 (32.5)	30 (40)	40 (37.25)	40 (30)	.272
Pulmonary function, (Mean ± SD)					
FEV1	1.9 ± 0.6 *,#,&	1.6 ± 0.6 ♠	1.5 ± 0.6 ▲	1.3 ± 0.5	**<.001**
FEV1 %pred	73.7 ± 18.9 *,#,&	64.2 ± 20.5 ♠	58.7 ± 19.3 ▲	51.9 ± 16.2	**<.001**
FVC	3.2 ± 0.8 #,&	3.0 ± 0.8 ♠	2.9 ± 0.7	2.8 ± 0.6	**<.001**
FEV1/FVC	57.8 ± 10.0 *,#,&	53.2 ± 10.1 ♠	50.7 ± 10.7 ▲	46.6 ± 10.3	**<.001**
PEF	5.0 ± 1.8 *,#,&	4.3 ± 1.8 ♠	3.9 ± 1.6	3.6 ± 1.4	**<.001**
GOLD grades, *n* (%)					**<.001**
1	121 (34.8) *,#,&	25 (22.7) ♦ ♠	15 (12.3) ▲	6 (3.6)	
2	192 (55.2)	56 (50.9)	63 (51.6)	84 (50.6)	
3	33 (9.4) *,#,&	24 (21.8) ♦ ♠	40 (32.8) ▲	66 (39.8)	
4	2 (0.6) *,#,&	5 (4.6) ♦	4 (3.3) ▲	10 (6.0)	
GOLD group, *n* (%)					.773
Group A	235 (67.5)	75 (68.2)	82 (67.2)	105 (63.3)	
Group C	113 (32.5)	35 (31.8)	40 (32.8)	61 (36.7)	
CAT, (Mean ± SD)	8.3 ± 4.0 *,&	10.3 ± 4.7	9.1 ± 4.7	10.0 ± 4.9	**<.001**
mMRC, (Median, IQR)	1 (0) #,&	1 (0)	1 (1)	1 (1)	**<.001**
CCQ, (Mean ± SD)	15.2 ± 6.0 *,&	17.0 ± 6.1 ♠	16.4 ± 5.4 ▲	18.0 ± 6.6	**<.001**
Exacerbations in the past year, (Median, IQR)	0 (1)	0 (1)	0 (1)	0 (1)	.806
Exacerbations in the past year, *n* (%)					.983
0	202 (58.1)	62 (56.3)	68 (55.7)	91 (54.8)	
1	91 (26.1)	29 (26.4)	31 (25.4)	44 (26.5)	
≥2	55 (15.8)	19 (17.3)	23 (18.9)	31 (18.7)	
Hospitalizations in the past year, (Median, IQR)	0 (1)	0 (0)	0 (0)	0 (1)	0.753
Hospitalizations in the past year, *n* (%)					.567
0	260 (74.7)	84 (76.4)	94 (77.1)	117 (70.5)	
≥1	88 (25.3)	26 (23.6)	28 (22.9)	49 (29.5)	

Notes: The bold P values indicate statistical significance.

Abbreviations: BMI: Body Mass Index; CAT: COPD Assessment Test; CCQ: Clinical COPD Questionnaire; FEV1: Forced Expiratory Volume in one second; FVC: Forced Vital Capacity; GOLD: Global Initiative for Chronic Obstructive Lung Disease; ICS: Inhaled Corticosteroid; LAMA: Long-Acting Muscarinic Antagonist; LABA: Long-Acting β2-Agonist; IQR: Interquartile Range; mMRC: modified Medical Research Council; PEF: Peak Expiratory Flow; SD: Standard Deviation.

*Compared with the LABA + ICS group, *p* < .05.

#♦ Compared with the LABA + LAMA group, *p* < .05.

&♠ ▲ Compared with the LABA + LAMA + ICS group, *p* < .05.

### Future exacerbations and mortality of different inhalation therapies on less symptomatic patients

After 1 year of follow-up, there were no significant differences in the number of exacerbations and hospitalizations among the inhalation therapy groups. In addition, the proportion of exacerbations (0, 1, and ≥2) and hospitalizations (0 and ≥1) showed no significant differences. The mortality in the LAMA, LABA + ICS, LABA + LAMA, and LABA + LAMA + ICS groups were 1.3%, 1.1%, 0%, and 0.7%, respectively. There were no significant differences among the groups (Table 4).

**Table 4.** Exacerbation and mortality during one year of follow-up of different inhalation therapies on less symptomatic COPD patients.

Variables	LAMA (*n* = 307)	LABA + ICS (*n* = 91)	LABA + LAMA (*n* = 101)	LABA + LAMA + ICS (*n* = 138)	*p*-value
Exacerbations, (Median, IQR)	0 (1)	0 (1)	0 (1)	0 (1)	.436
Exacerbations, *n* (%)					.583
0	203 (67.0)	65 (72.2)	63 (62.4)	90 (65.7)	
1	61 (20.1)	15 (16.7)	22 (21.8)	34 (24.8)	
≥2	39 (12.9)	10 (11.1)	16 (15.8)	13 (9.5)	
Hospitalizations, (Median, IQR)	0 (0)	0 (0)	0 (0)	0 (0)	.304
Hospitalizations, *n* (%)					.165
0	247 (81.5)	80 (88.9)	80 (79.2)	106 (77.4)	
≥1	56 (18.5)	10 (11.1)	21 (20.8)	31 (22.6)	
Mortality, *n* (%)	4 (1.3)	1 (1.1)	0 (0)	1 (0.7)	.686

Abbreviations: COPD: Chronic Obstructive Pulmonary Disease; IQR: Interquartile Range; ICS: Inhaled Corticosteroid; LAMA: Long-Acting Muscarinic Antagonist; LABA: Long-Acting β2-Agonist.

After adjusted for age, sex, BMI, education level, smoke history, CAT, mMRC, CCQ, pulmonary function and exacerbation in the past year, the future exacerbations and mortality showed no significant differences among the groups in less symptomatic patients (Supplemental Table 1).

### Future exacerbations and mortality of different inhalation therapies in Groups A and C patients

Furthermore, we analyzed the future exacerbations and mortality among different inhalation therapies in Groups A and C patients. After 1 year of follow-up, there were no significant differences in the number of exacerbations and hospitalizations among different inhalation therapies in Groups A and C. In addition, the proportion of exacerbations (0, 1, and ≥ 2) and hospitalizations (0 and ≥ 1) showed no significant differences. Also, the mortality showed no significant differences among different inhalation therapies (Table 5).

**Table 5.** Exacerbation and mortality during one year of follow-up of different inhalation therapies on Groups A and C patients.

	Group A (*N* = 419)					Group C (*N* = 218)				*p*-value
Variables	LAMA (*n* = 207)	LABA + ICS (*n* = 59)	LABA + LAMA (*n* = 69)	LABA + LAMA + ICS (*n* = 84)	*p*-value	LAMA (*n* = 100)	LABA + ICS (*n* = 32)	LABA + LAMA (*n* = 32)	LABA + LAMA + ICS (*n* = 54)	
Exacerbations, (Median, IQR)	0 (1)	0 (0)	0 (1)	0 (0)	0.657	0 (1)	0 (1.75)	1 (1.75)	0.5 (1)	.853
Exacerbations, *n* (%)					0.882					.532
0	152 (74.5)	45 (77.6)	48 (69.6)	63 (75.9)		51 (51.5)	20 (62.5)	15 (46.9)	27 (50.0)	
1	35 (17.2)	10 (17.2)	13 (18.8)	15 (18.1)		26 (26.3)	5 (15.6)	9 (28.1)	19 (35.2)	
≥2	17 (8.3)	3 (5.2)	8 (11.6)	5 (6.0)		22 (22.2)	7 (21.9)	8 (25.0)	8 (14.8)	
Hospitalizations, (Median, IQR)	0 (0)	0 (0)	0 (0)	0 (0)	0.657	0 (1)	0 (0)	0 (1)	0 (1)	.060
Hospitalizations, *n* (%)					0.664					.289
0	176 (86.3)	53 (91.4)	59 (85.5)	70 (84.3)		71 (71.7)	27 (84.4)	21 (65.6)	36 (66.7)	
≥1	28 (13.7)	5 (8.6)	10 (14.5)	13 (15.7)		28 (28.3)	5 (15.6)	11 (34.4)	18 (33.3)	
Mortality, *n* (%)	3 (1.4)	1 (1.7)	0 (0)	1 (1.2)	0.850	1 (1.0)	0 (0)	0 (0)	0 (0)	1.000

Abbreviations: IQR: Interquartile Range; ICS: Inhaled Corticosteroid; LAMA: Long-Acting Muscarinic Antagonist; LABA: Long-Acting β2-Agonist.

After adjusted for age, sex, BMI, education level, smoke history, CAT, mMRC, CCQ, pulmonary function and exacerbation in the past year, the future exacerbations and mortality showed no significant differences among the groups in Groups A and C patients (Supplemental Tables 2 and 3).

## Discussion

The initial therapy for Group A should be a bronchodilator based on its effect on breathlessness which can be a short or long-acting bronchodilator [12]. The initial therapy for Group C patients should be a LAMA. However, in this real-world study, we found that whether in Group A and Group C, less than 50% of the patients had been offered initial inhalation therapy adhered GOPD document; this finding is consistent with previous studies [13–15]. This finding also implies that in China, many clinicians do not completely follow the GOLD document to offer the initial inhalation therapy for less symptomatic patients with COPD. Therefore, it is critical to explore the possible causes as to why the GOLD document are not followed.

As LAMA, LABA/ICS, LABA/LAMA and LABA/ICS + LAMA were the top used inhalants in the real world, we analyzed the clinical characteristics of different inhalation therapies among less symptomatic patients with COPD. We found that pulmonary function was better in patients on LAMA mono-therapy when compared with other groups. These findings indicate that clinicians offer initial inhaled prescriptions for less symptomatic patients based on pulmonary function parameters. In other words, the patient’s pulmonary function results might be one reason why clinicians do not follow the GOLD document for the initial inhalation therapy in less symptomatic COPD patients.

Currently, most randomized controlled trials (RCTs) or meta-analyses on the effectiveness of single, double, or triple inhaled drugs has just focused on all patients with COPD rather than examining drug effectiveness according to the GOLD groups [16–17]. In addition, more symptomatic patients with COPD have accounted for the majority of the study populations [8,18], and studies have shown that more symptomatic patients with COPD treated with LABA + LAMA or LABA + LAMA + ICS are more likely to attain a minimum clinically important difference than patients treated with a LAMA [19]. In fact, in China, patients with COPD typically do not go to the hospital until they have severe respiratory symptoms. So, there are quite fewer patients in the less symptomatic group. Therefore, clinicians seem to have overlooked different inhalation therapies and RCTs for less symptomatic patients.

With this real-world study, we are the first to analyze future exacerbations and mortality on less symptomatic COPD patients with different initial inhalation therapies during 1 year of follow-up. Interestingly, the number of exacerbations and hospitalizations as well as mortality were not significantly different among single, double and triple-inhaled drugs. Furthermore, there were no significant differences in Groups A and C patients. Although there was study showed that COPD patients with frequent exacerbations treated with triple inhaled therapy combining fluticasone furoate, umeclidinium and vilanterol could significantly decrease future exacerbation, improve airflow limitation and lung hyperinflation [20] which was not consistent with our results. The main reason may be that our study focused on less symptomatic COPD patients. Comprehensively, even though the GOLD 2019 document separated pulmonary function from the combined assessment tool, clinicians still offer initial inhaled prescriptions for less symptomatic patients based on pulmonary function parameters in the real word. In fact, using pulmonary function results to guide the initial inhaled therapies for less symptomatic patients with COPD does not lead to better outcomes (including exacerbations and mortality). In other words, the clinicians should offer a single inhaled drug of LAMA to less symptomatic COPD patients and regardless of the pulmonary function results. However, this is just a real-world study, RCTs or meta-analyses of different inhalation therapies are necessary for less symptomatic patients with COPD in the future.

This study has some limitations. First, some of the patients stopped/changed drugs therapy while most of the patients remained stable during one year of follow-up. However, we have compared the number of exacerbations and hospitalizations between the patients who remained stable and those who stopped/changed drugs therapy; there were no significant differences (Supplemental Table 4). Then, a total of 109 patients lost to follow-up, but we have analyzed the characteristics of these patients and found that there were no significant differences (Supplemental Table 5). Moreover, the results of this study were related to the Chinese population only and thus more national population was needed in the future. Furthermore, because this was only a real-world study, we did not design a questionnaire specifically to investigate the specific reasons why clinicians did not offer initial inhalation therapy according to the GOLD document. This will be our future research direction. Finally, the patients in Group A accounted for the majority, while patients in Group C were small. In addition, the patients just followed up for one year to observe the future exacerbations and mortality in this study.

## Conclusions

There was a significant discrepancy between the recommendations of the GOLD document and the real-world clinical practice in a Chinese population on less symptomatic COPD patients. Over half of the less symptomatic patients received inhalation therapy that were inconsistent with the GOLD document recommendations. In addition, less symptomatic COPD patients treated with LAMA mono-therapy had better pulmonary function. However, the future exacerbations and mortality were not significantly different among different inhalation therapies groups. These findings indicate that the single inhaled drug of LAMA should be recommended and pulmonary function is not a good indicator for the choice of initial inhalation therapy in less symptomatic COPD patients.

## Supplementary Material

Supplemental Material

## Data Availability

All data of this study are available from the corresponding author Ping Chen for reasonable request.
