# Supplementary material for: The effects of different inhalation therapies on less symptomatic chronic obstructive pulmonary disease patients in a Chinese population: a real-world study
Source: Ann Med. 2023 Mar 29;55(1):1317–24. doi: 10.1080/07853890.2023.2192519 (PMC10062218; doi:10.1080/07853890.2023.2192519)
Supplement: Supplemental Material [file IANN_A_2192519_SM0577.docx]

**Supplemental table 1. Multivariate analysis for future exacerbations and mortality on less symptomatic patients.**

| **Variables** | **Exacerbation** | | | | **Mortality** | | | |
| --- | --- | --- | --- | --- | --- | --- | --- | --- |
|  | **OR (95%CI)** | **P-value** | **adj. OR (95%CI)** | **adj. P-value** | **OR (95%CI)** | **P-value** | **adj. OR (95%CI)** | **adj. P-value** |
| Therapy |  |  |  |  |  |  |  |  |
| LAMA | Reference |  | Reference |  | Reference |  | Reference |  |
| LABA+ICS | 0.78 (0.46-1.31) | 0.351 | 0.76 (0.44-1.32) | 0.331 | 0.84 (0.09-7.63) | 0.878 | 0.86 (0.04-16.83) | 0.923 |
| LABA+LAMA | 1.22 (0.77-1.96) | 0.397 | 1.36 (0.81-2.27) | 0.244 | 0 (0-Inf) | 0.993 | 0 (0-Inf) | 0.996 |
| LABA+LAMA+ICS | 1.03 (0.67-1.57) | 0.906 | 1.12 (0.68-1.84) | 0.655 | 0.55 (0.06-4.99) | 0.598 | 1.07 (0.07-16.27) | 0.962 |
| Age | 1.01 (0.99-1.03) | 0.439 | 0.99 (0.96-1.01) | 0.332 | 1.12 (1.01-1.23) | 0.029 | 1.07 (0.93-1.23) | 0.328 |
| Sex |  |  |  |  |  |  |  |  |
| Male | Reference |  | Reference |  | Reference |  | Reference |  |
| Female | 1.01 (0.61-1.69) | 0.967 | 0.56 (0.23-1.39) | 0.213 | 1.48 (0.17-12.86) | 0.721 | 0.07 (0.00-6.21) | 0.241 |
| BMI | 0.99 (0.93-1.04) | 0.583 | 0.97 (0.91-1.03) | 0.281 | 0.93 (0.71-1.21) | 0.590 | 0.84 (0.60-1.16) | 0.285 |
| Education level |  |  |  |  |  |  |  |  |
| Primary school | Reference |  | Reference |  | Reference |  | Reference |  |
| Junior high school | 1.13 (0.77-1.67) | 0.536 | 1.38 (0.90-2.12) | 0.143 | 0 (0-Inf) | 0.993 | 0 (0-Inf) | 0.995 |
| High school | 1.05 (0.65-1.69) | 0.834 | 1.35 (0.81-2.26) | 0.246 | 1.36 (0.22-8.26) | 0.737 | 1.98 (0.22-18.20) | 0.545 |
| University | 1.35 (0.73-2.48) | 0.333 | 1.75 (0.90-3.42) | 0.100 | 1.45 (0.15-14.19) | 0.750 | 4.10 (0.22-74.90) | 0.341 |
| Smoke history |  |  |  |  |  |  |  |  |
| Never-smoker | Reference |  | Reference |  | Reference |  | Reference |  |
| Former-smoker | 0.89 (0.53-1.50) | 0.667 | 0.67 (0.31-1.55) | 0.442 | 0.31 (0.03-3.62) | 0.360 | 0.09 (0..00-3.00) | 0.179 |
| Current-smoker | 0.85 (0.54-1.34) | 0.484 | 0.76 (0.37-1.55) | 0.442 | 0.42 (0.07-2.54) | 0.344 | 0.23 (0.01-4.19) | 0.318 |
| Pulmonary function |  |  |  |  |  |  |  |  |
| FEV1 | 0.99 (0.76-1.30) | 0.953 | 0.51 (0.11-2.26) | 0.374 | 1.38 (0.39-4.80) | 0.617 | 0.35 (1.00-156.27) | 0.734 |
| FEV1 %pred | 1.00 (1.00-1.01) | 0.408 | 1.03 (1.00-1.05) | 0.170 | 1.04 (1.01-1.08) | 0.020 | 1.06 (0.98-1.14) | 0.151 |
| FVC | 0.97 (0.78-1.21) | 0.787 | 1.16 (0.57-2.35) | 0.680 | 0.85 (0.29-2.49) | 0.769 | 0.85 (0.04-17.46) | 0.916 |
| FEV1/FVC | 1.00 (0.99-1.02) | 0.803 | 1.01 (0.97-1.05) | 0.678 | 1.05 (0.97-1.15) | 0.232 | 1.03 (0.92-1.15) | 0.624 |
| PEF | 0.98 (0.89-1.07) | 0.642 | 0.91 (0.75-1.12) | 0.386 | 1.03 (0.66-1.61) | 0.891 | 1.08 (0.37-3.20) | 0.883 |
| CAT | 0.98 (0.94-1.02) | 0.241 | 0.96 (0.92-1.01) | 0.121 | 0.96 (0.78-1.17) | 0.683 | 0.97 (0.71-1.31) | 0.822 |
| mMRC | 1.27 (1.00-1.60) | 0.046 | 1.21 (0.92-1.01) | 0.169 | 1.06 (0.35-3.27) | 0.913 | 1.69 (0.33-8.73) | 0.531 |
| CCQ | 1.02 (0.99-1.05) | 0.169 | 1.02 (0.99-1.06) | 0.209 | 1.02 (0.99-1.05) | 0.169 | 1.02 (0.99-1.06) | 0.209 |
| Exacerbations in the past year | 1.19 (1.07-1.34) | 0.002 | 1.07 (0.94-1.22) | 0.302 | 1.02 (0.90-1.16) | 0.745 | 1.07 (0.88-1.30) | 0.510 |

**Notes:** adj. P-value, after adjusted for variables of age, sex, BMI, education level, smoke history, CAT, mMRC, CCQ, pulmonary function and exacerbation in the past year.

**Abbreviations:** BMI, Body Mass Index; CI, Confidence Interval; CAT, COPD Assessment Test; CCQ, Clinical COPD Questionnaire; FEV1, Forced Expiratory Volume in one second; FVC, Forced Vital Capacity; ICS, Inhaled Corticosteroid; LAMA, Long-Acting Muscarinic Antagonist; LABA, Long-Acting β2-Agonist; mMRC, modiﬁed Medical Research Council; PEF, Peak Expiratory Flow; OR, Odds Ratio.

.

**Supplemental table 2. Multivariate analysis for future exacerbations and mortality on Group A patients.**

| **Variables** | **Exacerbation** | | | | **Mortality** | | | |
| --- | --- | --- | --- | --- | --- | --- | --- | --- |
|  | **OR (95%CI)** | **P-value** | **adj. OR (95%CI)** | **adj. P-value** | **OR (95%CI)** | **P-value** | **adj. OR (95%CI)** | **Adj. P-value** |
| Therapy |  |  |  |  |  |  |  |  |
| LAMA | Reference |  | Reference |  | Reference |  | Reference |  |
| LABA+ICS | 0.86 (0.43-1.73) | 0.679 | 0.84 (0.40-1.74) | 0.631 | 1.19 (0.12-11.69) | 0.880 | 0.59 (0.01-31.25) | 0.792 |
| LABA+LAMA | 1.28 (0.70-2.33) | 0.423 | 1.39 (0.70-2.74) | 0.344 | 0 (0-Inf) | 0.994 | 0 (0-Inf) | 0.997 |
| LABA+LAMA+ICS | 0.87 (0.48-1.58) | 0.644 | 1.05 (0.53-2.11) | 0.883 | 0.82 (0.08-7.99) | 0.864 | 2.20 (0.06-78.74) | 0.665 |
| Age | 0.99 (0.97-1.01) | 0.383 | 0.98 (0.95-1.02) | 0.395 | 1.14 (1.02-1.27) | 0.017 | 1.05 (0.89-1.24) | 0.573 |
| Sex |  |  |  |  |  |  |  |  |
| Male | Reference |  | Reference |  | Reference |  | Reference |  |
| Female | 0.98 (0.47-2.01) | 0.946 | 0.52 (0.15-1.84) | 0.314 | 2.10 (0.23-19.18) | 0.512 | 0.01 (0.00-3.07) | 0.113 |
| BMI | 0.97 (0.90-1.04) | 0.407 | 0.96 (0.89-1.04) | 0.333 | 0.94 (0.70-1.25) | 0.655 | 0.65 (0.35-1.20) | 0.165 |
| Education level |  |  |  |  |  |  |  |  |
| Primary school | Reference |  | Reference |  | Reference |  | Reference |  |
| Junior high school | 1.41 (0.83-2.39) | 0.202 | 1.51 (0.84-2.72) | 0.171 | 0 (0-Inf) | 0.994 | 0 (0-Inf) | 0.996 |
| High school | 1.05 (0.55-1.98) | 0.889 | 1.10 (0.55-2.17) | 0.791 | 0.62 (0.06-6.04) | 0.679 | 0.71 (0.02-31.64) | 0.860 |
| University | 1.47 (0.66-3.27) | 0.348 | 1.35 (0.54-3.36) | 0.519 | 1.37 (0.14-13.54) | 0.788 | 1.56 (0.03-69.80) | 0.818 |
| Smoke history |  |  |  |  |  |  |  |  |
| Never-smoker | Reference |  | Reference |  | Reference |  | Reference |  |
| Former-smoker | 0.71 (0.33-1.51) | 0.371 | 0.56 (0.18-1.72) | 0.313 | 0.32 (0.03-3.64) | 0.360 | 0.03 (0..00-2.49) | 0.120 |
| Current-smoker | 1.01 (0.54-1.91) | 0.968 | 0.87 (0.32-2.37) | 0.782 | 0.23 (0.03-1.67) | 0.147 | 0.02 (0.00-2.38) | 0.111 |
| Pulmonary function |  |  |  |  |  |  |  |  |
| FEV1 | 1.07 (0.75-1.54) | 0.705 | 0.38 (0.04-3.64) | 0.399 | 0.99 (0.23-4.21) | 0.989 | 1.40 (0.00-16.27) | 0.931 |
| FEV1 %pred | 1.00 (0.99-1.01) | 0.760 | 1.02 (0.98-1.05) | 0.308 | 1.05 (1.01-1.09) | 0.024 | 1.03 (0.99-1.29) | 0.073 |
| FVC | 1.09 (0.81-1.47) | 0.550 | 1.60 (0.49-5.16) | 0.434 | 0.74 (0.22-2.51) | 0.634 | 0.36 (0.01-22.04) | 0.626 |
| FEV1/FVC | 1.00 (0.98-1.02) | 0.753 | 1.03 (0.95-1.11) | 0.474 | 1.03 (0.94-1.12) | 0.574 | 0.85 (0.61-1.19) | 0.348 |
| PEF | 1.02 (0.90-1.15) | 0.804 | 0.90 (0.69-1.18) | 0.445 | 0.87 (0.51-1.50) | 0.619 | 0.90 (0.19-4.30) | 0.897 |
| CAT | 0.97 (0.92-1.02) | 0.263 | 0.95 (0.88-1.02) | 0.128 | 0.97 (0.78-1.20) | 0.775 | 0.93 (0.65-1.33) | 0.674 |
| mMRC | 1.01 (0.74-1.38) | 0.955 | 1.08 (0.75-1.55) | 0.691 | 0.85 (0.24-3.01) | 0.796 | 1.07 (0.13-8.63) | 0.947 |
| CCQ | 1.01 (0.97-1.05) | 0.568 | 1.03 (0.98-1.08) | 0.184 | 1.02 (0.88-1.17) | 0.812 | 1.01 (0.77-1.31) | 0.970 |
| Exacerbations in the past year | 2.70 (1.51-4.81) | 0.001 | 2.82 (1.52-5.22) | 0.001 | 1.56 (0.17-14.22) | 0.693 | 5.82 (0.30-114.16) | 0.246 |

**Notes:** adj. P-value, after adjusted for variables of age, sex, BMI, education level, smoke history, CAT, mMRC, CCQ, pulmonary function and exacerbation in the past year.

**Abbreviations:** BMI, Body Mass Index; CI, Confidence Interval; CAT, COPD Assessment Test; CCQ, Clinical COPD Questionnaire; FEV1, Forced Expiratory Volume in one second; FVC, Forced Vital Capacity; ICS, Inhaled Corticosteroid; LAMA, Long-Acting Muscarinic Antagonist; LABA, Long-Acting β2-Agonist; mMRC, modiﬁed Medical Research Council; PEF, Peak Expiratory Flow; OR, Odds Ratio.

**Supplemental table 3. Multivariate analysis for future exacerbation and mortality on Group C patients.**

| **Variables** | **Exacerbation** | | | | **Mortality** | | | |
| --- | --- | --- | --- | --- | --- | --- | --- | --- |
|  | **OR (95%CI)** | **P-value** | **adj. OR (95%CI)** | **adj. P-value** | **OR (95%CI)** | **P-value** | **adj. OR (95%CI)** | **adj. P-value** |
| Therapy |  |  |  |  |  |  |  |  |
| LAMA | Reference |  | Reference |  | N/A |  | N/A |  |
| LABA+ICS | 0.61 (0.27-1.37) | 0.228 | 0.63 (0.26-1.54) | 0.314 | N/A | N/A | N/A | N/A |
| LABA+LAMA | 1.20 (0.54-2.68) | 0.648 | 1.59 (0.64-3.94) | 0.318 | N/A | N/A | N/A | N/A |
| LABA+LAMA+ICS | 1.06 (0.55-2.06) | 0.858 | 1.26 (0.57-2.79) | 0.561 | N/A | N/A | N/A | N/A |
| Age | 1.03 (1.00-1.06) | 0.087 | 1.00 (0.95-1.05) | 0.911 | N/A | N/A | N/A | N/A |
| Sex |  |  |  |  |  |  |  |  |
| Male | Reference |  | Reference |  | N/A |  | N/A |  |
| Female | 0.89 (0.41-1.90) | 0.759 | 0.60 (0.14-2.62) | 0.496 | N/A | N/A | N/A | N/A |
| BMI | 1.01 (0.92-1.09) | 0.908 | 1.03 (0.93-1.13) | 0.604 | N/A | N/A | N/A | N/A |
| Education level |  |  |  |  |  |  |  |  |
| Primary school | Reference |  | Reference |  | N/A |  | N/A |  |
| Junior high school | 0.87 (0.47-1.60) | 0.649 | 0.98 (0.49-1.99) | 0.964 | N/A | N/A | N/A | N/A |
| High school | 1.33 (0.61-2.93) | 0.475 | 1.78 (0.73-4.36) | 0.206 | N/A | N/A | N/A | N/A |
| University | 1.40 (0.51-3.89) | 0.516 | 2.45 (0.73-8.18) | 0.145 | N/A | N/A | N/A | N/A |
| Smoke history |  |  |  |  |  |  |  |  |
| Never-smoker | Reference |  | Reference |  | N/A |  | N/A |  |
| Former-smoker | 1.11 (0.52-2.40) | 0.785 | 1.03 (0.29-3.60) | 0.963 | N/A | N/A | N/A | N/A |
| Current-smoker | 0.86 (0.42-1.74) | 0.665 | 0.88 (0.28-2.82) | 0.836 | N/A | N/A | N/A | N/A |
| Pulmonary function |  |  |  |  |  |  |  |  |
| FEV1 | 1.14 (0.73-1.77) | 0.560 | 0.68 (0.04-10.90) | 0.783 | N/A | N/A | N/A | N/A |
| FEV1 %pred | 1.01 (1.00-1.02) | 0.086 | 1.05 (1.00-1.10) | 0.059 | N/A | N/A | N/A | N/A |
| FVC | 1.01 (0.72-1.43) | 0.934 | 0.98 (0.28-3.47) | 0.974 | N/A | N/A | N/A | N/A |
| FEV1/FVC | 1.01 (0.99-1.03) | 0.469 | 0.99 (0.93-1.06) | 0.761 | N/A | N/A | N/A | N/A |
| PEF | 1.01 (0.87-1.17) | 0.946 | 0.80 (0.57-1.13) | 0.204 | N/A | N/A | N/A | N/A |
| CAT | 0.95 (0.89-1.01) | 0.129 | 0.97 (0.90-1.05) | 0.508 | N/A | N/A | N/A | N/A |
| mMRC | 1.55 (1.05-2.29) | 0.027 | 1.67 (1.04-2.69) | 0.033 | N/A | N/A | N/A | N/A |
| CCQ | 1.00 (0.96-1.05) | 0.904 | 1.01 (0.95-1.08) | 0.693 | N/A | N/A | N/A | N/A |
| Exacerbations in the past year | 0.90 (0.77-1.05) | 0.168 | 0.87 (0.73-1.04) | 0.128 | N/A | N/A | N/A | N/A |

**Notes:** adj. P-value, after adjusted for variables of age, sex, BMI, education level, smoke history, CAT, mMRC, CCQ, pulmonary function and exacerbation in the past year. N/A, Not Applicable.

**Abbreviations:** BMI, Body Mass Index; CI, Confidence Interval; CAT, COPD Assessment Test; CCQ, Clinical COPD Questionnaire; FEV1, Forced Expiratory Volume in one second; FVC, Forced Vital Capacity; ICS, Inhaled Corticosteroid; LAMA, Long-Acting Muscarinic Antagonist; LABA, Long-Acting β2-Agonist; mMRC, modiﬁed Medical Research Council; PEF, Peak Expiratory Flow; OR, Odds Ratio.

**Supplemental table 4. Exacerbation and mortality between the patients of pharmacological regimens remained stable and patients who stopped/changed drugs therapy during one year of follow-up.**

| **Variables** | **Less symptomatic patients (N=637)** | | **P -**  **value** |
| --- | --- | --- | --- |
|  | **A_1_ (n=537)** | **A_2_ (n=100)** |  |
| Exacerbations, (Median, IQR) | 0 (1) | 0 (1) | 0.863 |
| Exacerbations, n (%) |  |  | 0.831 |
| 0 | 355 (66.5) | 66 (68.0) |  |
| 1 | 113 (21.2) | 18 (18.6) |  |
| ≥2 | 66 (12.3) | 13 (13.4) |  |
| Hospitalizations, (Median, IQR) | 0 (0) | 0 (0) | 0.242 |
| Hospitalizations, n (%) |  |  | 0.241 |
| 0 | 430 (80.5) | 83 (85.6) |  |
| ≥1 | 104 (19.5) | 14 (14.4) |  |
| Mortality, n (%) | 3 (0.6) | 3 (3.0) | 0.052 |

**Notes:** A_1_, patients of pharmacological regimens remained stable; A_2_, patients who stop/changed inhaled drugs therapy.

**Abbreviations:** IQR, Interquartile Range.

**Supplemental table 5. The clinical characteristics on less symptomatic COPD patients who remained in the study and lost to follow-up.**

| **Variables** | **Less symptomatic patients (N=746)** | | **P *-***  **value** |
| --- | --- | --- | --- |
|  | **A_1_ (n=637)** | **A_2_ (n=109)** |  |
| Age (years), (Mean ± SD) | 63.0 ± 9.1 | 64.1 ± 8.8 | 0.246 |
| Sex, n (%) |  |  | 0.787 |
| Male | 561 (88.1) | 95 (87.2) |  |
| Female | 76 (11.9) | 14 (12.8) |  |
| Education level, n (%) |  |  | 0.202 |
| Primary school | 242 (38.0) | 31 (28.4) |  |
| Junior high school | 220 (34.5) | 45 (41.3) |  |
| High school | 119 (18.7) | 25 (22.9) |  |
| University | 56 (8.8) | 8 (7.4) |  |
| BMI (kg/m^2^), (Mean ± SD) | 23.1 ± 3.1 | 23.1 ± 3.9 | 0.998 |
| Smoke history, n (%) |  |  | 0.251 |
| Never-smoker | 105 (16.5) | 25 (22.9) |  |
| Ex-smoker | 160 (25.1) | 24 (22.0) |  |
| Current-smoker | 372 (58.4) | 60 (55.1) |  |
| Smoking, (pack/year) (Median, IQR) | 35 (31) | 33 (37.5) | 0.254 |
| Pulmonary function, (Mean ± SD) |  |  |  |
| FEV1 | 1.7 ± 0.6 | 1.5 ± 0.6 | 0.057 |
| FEV1 %pred | 65.1 ± 20.6 | 63.1 ± 20.8 | 0.287 |
| FVC | 3.0 ± 0.8 | 2.9 ± 0.7 | 0.081 |
| FEV1/FVC | 53.5 ± 11.1 | 53.0 ± 11.2 | 0.642 |
| PEF | 4.3 ± 1.8 | 4.1 ± 1.6 | 0.068 |
| GOLD grades, n (%) |  |  | 0.486 |
| 1 | 143 (22.4) | 24 (22.0) |  |
| 2 | 344 (54.0) | 52 (47.7) |  |
| 3 | 133 (20.9) | 29 (26.6) |  |
| 4 | 17 (2.7) | 4 (3.7) |  |
| GOLD group, n (%) |  |  | 0.237 |
| Group A | 419 (65.8) | 78 (71.6) |  |
| Group C | 218 (34.2) | 31 (28.4) |  |
| CAT, (Mean ± SD) | 9.3± 4.3 | 10.1 ± 5.4 | 0.105 |
| mMRC, (Median, IQR) | 1 (1) | 1 (0) | 0.622 |
| CCQ, (Mean ± SD) | 17.3 ± 6.1 | 17.8 ± 6.5 | 0.451 |
| Exacerbations in the past year,  (Median, IQR) | 0 (1) | 0 (1) | 0.974 |
| Hospitalizations in the past year,  (Median, IQR) | 0 (1) | 0 (0) | 0.420 |

**Notes:** A_1_: The COPD patients who remained in the study after one year of follow-up; A_2_: The COPD patients who lost to follow-up.

**Abbreviations:** BMI, Body Mass Index; COPD, Chronic obstructive pulmonary disease; CAT, COPD Assessment Test; CCQ, Clinical COPD Questionnaire; FEV1, Forced Expiratory Volume in one second; FVC, Forced Vital Capacity; GOLD, Global Initiative for Chronic Obstructive Lung Disease; IQR, Interquartile Range; mMRC, modiﬁed Medical Research Council; PEF, Peak Expiratory Flow; SD, Standard Deviation.
